# Death of O. C. Gibbs, M. D.

**Published:** 1871-08

**Authors:** A. Waterhouse

**Affiliations:** Jamestown, N. Y.


### Death of O. C. Gibbs, M. D.

Died at Frewsburg, N. Y., July 28th, 1871, O. C. Gibbs, M. D., aged 47 years. He was born in Windsor, Ohio, graduated at the Cleveland Medical College in 1848, and commenced the practice of his profession in Wayne, Ohio, soon after which he moved to Perry, in the same State, where he remained until 1856, when he removed to Frewsburg, at which place he was engaged in an extensive and successful practice nearly up to the time of his death, enjoying the confidence of his neighboring physicians and the community in which he lived, to a very high degree.

Dr. Gibbs was, for some time, associate editor of the Philadelphia Medical and Surgical Reporter, and articles from his pen have been published in the Buffalo Medical and Surgical Journal, and in several of the Medical journals of the past few years.

Jamestown, N. Y., August 15th. A. WATERHOUSE, M. D.

